# Supplementary material for: Mental Health Is a Family Affair—Systematic Review and Meta-Analysis on the Associations between Mental Health Problems in Parents and Children during the COVID-19 Pandemic
Source: Int J Environ Res Public Health. 2023 Mar 2;20(5):4485. doi: 10.3390/ijerph20054485 (PMC10001622; doi:10.3390/ijerph20054485)
Supplement: Supplementary file 1 [file ijerph-20-04485-s001.zip › Table S1.pdf]

Table S1: Search strategy in Web of Science (All Databases).

**Parental Mental Health**				**COVID-19**		**Child Mental Health**				**Publication Year**
			AND		AND				AND	
parent*<br>maternal<br>mother*<br>paternal<br>father*<br>caregiver	NEAR/3	mental illness<br>mental* ill*<br>mental disorder*<br>affective disorder*<br>mood disorder*<br>depress*<br>depressive disorder*<br>anxiety<br>anxiety disorder*<br>stress<br>distress		Covid<br>Corona*<br>SARS-CoV-2<br>pandemic		child*<br>adolescent*<br>teen*<br>youth<br>famil*	NEAR/3	wellbeing<br>well-being<br>mental health<br>psychological health<br>mental illness<br>mental* ill*<br>mental disorder*<br>affective disorder*<br>mood disorder*<br>depress*<br>depressive disorder*<br>anxiety<br>anxiety disorder*<br>stress<br>distress		2020-2022

** truncation symbol; within a column, search terms were connected with OR; search terms joined by the NEAR/3 operator have to be within three words of each other.*
