# Supplementary material for: Mental Health Is a Family Affair—Systematic Review and Meta-Analysis on the Associations between Mental Health Problems in Parents and Children during the COVID-19 Pandemic
Source: Int J Environ Res Public Health. 2023 Mar 2;20(5):4485. doi: 10.3390/ijerph20054485 (PMC10001622; doi:10.3390/ijerph20054485)
Supplement: Supplementary file 1 [file ijerph-20-04485-s001.zip › Table S2.pdf]

Table S2: Overview of included studies.

Study	Country	Survey Method	Study Design	Study Period (If Longitudinal)	Relevant COVID-19 Assessment Point	Study Population						Parent- or Child-/ Adolescent-Report
						Parents		Children				
						n	% male	n	% male	Age Range; *M* (*SD*)	Focus Children/ Adolesents	
Achterberg et al. (2021) [61]	Netherlands	online	longi	2015 – 2020	28.04. – 10.05.20	106	7	151	53.0	10.76 - 13.65; 12 (0.81)	adol	both
Akgül and Atalan Ergin (2021) [62]	Turkey	online	cross		nr	155	16.8	155	40	12 - 18; 14.63 (2.04)	adol	both
Andrés-Romero et al. (2021) [128]	Spain	online	cross		nr	883	6.1	1555	46.82	3 - 18; nr (nr)	both	parent
Babore et al. (2021) [28]	Italy	online	cross		04.2020	206	0	206	49.5	7 - 18; 12.18 (3.31)	both	both
Berry et al. (2021) [107]	Ireland	online	longi	28.03 - 21.10.20	28.03. - 18.05.20	159	6.9	159	nr	4 - 18; nr (nr)	both	parent
Bianco et al. (2021) [61]	Italy	online	cross		01.04. - 04.05.20	305	0	305	50.5	6 - 13; 10.3 (2.4)	both	parent
Black et al. (2021) [53]	USA	online	cross		nr	391	70	391	70	8 - 17; 10.68 (2.28)	both	both
Blackwell et al. (2022) [108]	USA	online + paper-pencil + phone interviews	cross		01. - 31.05.21	977<br>669	nr<br>nr	977<br>669	51.9<br>49.2	2 - 12, 8.3 (2.3)<br>11 - 17, 16.4 (1)	both	parent<br>both
Borbás et al. (2021) [64]	Switzerland	online	longi	03. - 05.20	03. - 05.20	21	0	26	61	7 - 17; 10.69 (2.52)	both	parent
Browne et al. (2021) [65]	UK (76 %), USA (19 %), Canada (4 %), Australia (1 %)	online	longi	05. - 07.2029	05.2020	549	29	1098	51.8	5 - 18; 10.71 (3.4)	both	parent
Büber and Aktaş Terzioğlu (2022) [109]	Turkey	online	Cross		03.07. - 31.08.20	1797	17.3	1797	50.1	6 - 12; 8.49 (0.28)	child	parent
Buechel et al. (2022) [66]	Germany	online	cross		02. - 06.2021	991 (sw)	6.3	437 (s2)	51.6 (sw)	1.4 - 3.1; 2.2 (.5) (s2)	child	parent
Chan (2022) [110]	Hong Kong	online	cross		05.2020	109	9.2	109	58.7	8 - 10; 8.98 (0.76)	child	both
Chartier et al. (2021) [111]	Belgium	online	cross		03. - 05.2020	287	20	161	54.72	8 - 18; 12 (3.82)	both	both
Cohodes et al. (2021) [112]	USA	online	cross		24. - 26.04.20	200	46	200	47.5	0.83 - 17; 8.84 (4.78)	both	parent
Corbett et al. (2021) [113]	USA	online	longi	2017 - 2020	04. - 05.2020	122	3.7	122	66.4	nr; nr (nr)	adol	both
Crescentini et al. (2020) [67]	Italy	online	cross		16.04 - 07.05.20	721	14.29	721	51.6	6 - 18; 10.08 (2.52)	both	parent
Cusinato et al. (2020) [129]	Italy	online	cross		25.04. - 08.05.20	463	9.5	nr	56.2	5 - 17; 9.72 (3.29)	both	parent
Daks et al. (2020) [68]	USA	online	cross		27.03. - 04.2020	742	29	nr	20	5 - 18; 9.4 (5.0)	both	parent
Davidson et al. (2021) [114]	USA	online	cross		22.04. - 22.05.20	286	7.7	nr	nr	nr; 6.21 (4.93)	child	parent
Doan et al. (2022) [54]	USA	online + lab	longi	2017 - 2020	05. - 08.2020	99	0	99	nr	nr; nr (nr)	child	parent
Dollberg and Hanetz-Gamliel (2022) [69]	Israel	online	cross		03. - 04.2020	152	0	152	48	3 - 12.66; 7.08 (2.54)	child	parent

Donker et al. (2021) [116]	Netherlands	nr	longi	fall 2019 - spring 2020	spring 2020	195	15 (in 2019)	190	49.2 (in 2019)	nr; 11.4 (.5) (in 2019)	both	both
Dubois-Comtois et al. (2021) [70]	Canada	phone-based survey	cross		18.04. - 18.05-20	144	9	144	51.4	9 - 12; 10.44 (1.09)	child	both
Essler et al. (2021) [117]	Germany	online	longi	04. - 07.2020	04. - 05.2020	2921	nr	nr	nr	3 - 10; nr	child	parent
Feinberg et al. (2022) [71]	USA	online	longi	2017 - 2020	04. - 05.2020	206)	40.8	297	54.4	nr; 9.9 (1)	child	parent
Ferraro et al. (2021) [72]	Italy	online	cross		23.05. - 24.06.21	221	3.2	246	nr	0 - 18; nr (nr)	child	parent
Feurer et al. (2021) [55]	USA	lab	longi	before COVID-19 - 09.2020	06. - 09.2020	45	0	45	17.8	9 - 16; 12.42 (2.31) (before COVID-19)	both	both
Fogarty et al. (2022) [56]	Australia	online	longi	04.2003 - 09.2020	06. - 09.2020	257	0	257	45.5	14 - 17; 15.47 (0.87)	adol	both
Foley et al. (2021) [73]	Australia, China, Italy, Sweden, UK, USA	online	cross		01.04. - 07.07.20	2516	8	2516	52.1	3 - 8; 5.77 (1.1)	child	parent
Fong et al. (2021) [74]	Malaysia	online	cross		07. - 10.2020	62	14.5	62	59.7	5 - 17; 8.7 (2.8)	both	parent
Fosco et al. (2022) [57]	USA	online	longi	2017 - 05.2020	05.2020	204	25	> 204	54.9	nr; 4.17 (2.17)	child	parent
Frigerio et al. (2022) [75]	Italy	online	longi	2017 - 06.2020	22.04. - 03.06.2020	74	0	59	54.2	4; 4.2 (.61)	child	parent
Giannotti et al. (2021) [130]	Italy	online	cross		20.4. - 18.5.2020	602	12.6	602	47.3	3 - 11; 7.99 (3.68)	both	parent
Giannotti et al. (2022) [131]	Spain	online	cross		03. - 06.2020	432	0	432	52.5	3 - 17; 6.30 (3)	both	parent
Glynn et al. (2021) [76]	USA	online	longi	before COVID-19 - 09.07.2020	05.05. - 09.07.2020	169	0	169	53.3	2.6 - 6; 4.1 (.93)	child	parent
Hails et al. (2022) [77]	USA	online	cross		07.2020 - 01.2021	267	8	267	nr	1.5 - 5.8; 3.43 (1.2)	child	parent
Hollenstein et al. (2021) [58]	Canada	lab (T1) + online (T2)	longi	09.2019 - 06.2021	15.05. - 25.06.20	155	0	146	55 (T1)	12 - 13; 12.49 (nr) (in 2019)	adol	both
Khoury et al. (2021) [78]	Canada	online	longi	2016 - 11.2020	05. - 11.2020	68	0	68	51.5	7 - 9; 7.87 (0.75)	child	parent
Kim et al. (2021) [79]	Korea	online	cross		01. - 30.06.20	217	nr	217	56.2	7 - 12; 9.16 (1.43)	child	parent
Köhler-Dauner et al. (2022) [59]	Germany	online	longi	10.2013 - 07.2020	18.05. - 31.07.20	91	0	91	52.7	5 - 7; 6.03 (.61)	child	parent
Lee et al. (2021) [80]	USA	online	cross		02. - 30.04.20	405	31	405	nr	0 - 12; nr (nr)	child	parent
Lengua et al. (2022) [60]	USA	university (T1) + online (T2 + T3)	longi	2018 - 01.2021	04. - 05.2020	143	0	143	56.6	nr; 14.33 (.48)	adol	both
Li et al. (2021) [81]	China	online	cross		02. - 03.2020	8483	23.24	8483	45.77	nr; 15.31 (0.018)	adol	both
Liang et al. (2021) [118]	Italy	online	longi	03. - 05.2020	03.2020	1053	4.5	288	54.9	11 - 18; 13.89 (2.15)	adol	parent
Lionetti et al. (2022) [132]	Italy	nr	longi	nr - 06.2020	04.2020	72	0	72	57	2 - 6; 3.82 (1.38)	child	parent
Low and Mounts (2022) [82]	USA	online	cross		06. - 11.2020	272	25.6	272	53.6	12 - 18; 14.74 (1.8)	adol	parent
Maggio et al. (2021) [83]	Italy	online	cross		17.03. - 02.05.21	96	5.2	96	48.9	5 - 16; 11.81 (3.23)	both	both
Marchetti et al. (2020) [84]	Italy	online	cross		03. - 14.04.20	878	12.6	878	51.7	3 - 13; 7.54 (3.16)	child	parent
Marzilli et al. (2021) [85]	Italy	online	cross		10.10.20 - 15.01.21	353	36	353	49.3	2 - 16; 9.28 (4.54)	both	parent

McArthur et al. (2021) [86]	Canada	nr	longi	2017 - 08.2020	05. - 08.2020	846	0	846	52.8	9 - 11; 9.85 (0.78)	child	both
McMahon et al. (2021) [87]	Ireland	online	cross		04. - 05.2020	790	8.2	797	54	4 - 12; 9 (nr)	child	parent
Mensi et al. (2021) [133]	Italy	online	cross		nr	1262	nr	1262	30.35	nr; 16.27 (1.63)	adol	adol
Morban et al. (2020) [88]	Dominican Republic	online	cross		27.04. - 11.05.20	29	10.3	nr	nr	1.5 - 5; nr (nr)	child	parent
Moulin et al. (2021) [89]	France	online	longi	2009 - 04.2020	24.03. - 28.04.20	432	34.9	432	51	nr; 6.8 (4.1)	child	parent
Orgilés et al. (2021) [119]	Italy, Spain, Portugal	online	cross		nr	515	8.6	515	54.2	3 - 18; 8.98 (4.29)	both	parent
Penner et al. (2022) [90]	USA	online	cross		02.02. - 04.04.21	796	51.8	796	57.9	5 - 16, 10.35, (3.16)	both	parent
Polónyiová et al. (2022) [91]	Slovakia	online	longi	06. - 12.2020	06. - 07.2020	177	nr	177	54.8	nr; 8.98 (nr)	child	parent
Radanović et al. (2021) [92]	Serbia	online	cross		16.04. - 06.05.20	376	nr	376	40.4	7 - 19; 12.77 (3.57)	both	both
Rizeq et al. (2021) [93]	Canada	online	longi	05. - 11.2020	05. - 09.2020	1069	nr	1069	57.34	nr; 11.06 (3.37)	both	both
Robertson et al. (2021) [94]	USA	online + paper pencil	longi	22.04 - 17.07.20	22.04 - 22.05.20	286	7.7	286	nr	nr; 6.21 (4.93)	child	parent
Romero et al. (2020) [95]	Spain	online	cross		08. - 27.04.20	1049	9.7	1049	49.6	3 - 12; 7.29 (2.39)	child	parent
Russell et al. (2020) [96]	USA	online	cross		27. - 28.04.20	420	51.9	420	nr	0 - 18; nr (nr)	both	parent
Russell et al. (2022) [120]	USA	online	longi	27.04 - 30.05.20	27. - 28.04.20	271	54.2	271	nr	0 - 18; nr (nr)	both	parent
Saddik et al. (2021) [97]	United Arab Emirates	online	cross		24.03 - 25.05.20	1054	nr	1160	nr	nr; nr (nr)	both	parent
Shelleby et al. (2022) [98]	USA	online	longi		20.04. - 20.06.2020	308	0	308	nr	nr; nr (nr)	child	parent
Singletary et al. (2022) [121]	USA	online	cross		07.05. - 20.06.20	559	3.6	559	56.4	0 - 9; 6.0 (2.6)	child	parent
Spencer et al. (2021) [99]	USA	online + telephone	longi	09.2019 - 01.2021	08.2020 - 01.2021	168	nr	168	58	5 - 11; 8.5 (1.8)	child	parent
Spinelli et al. (2020) [122]	Italy	online	cross		02. - 07.04.20	854	9.3	854	6.7	2 - 14; 7.14 (3.38)	child	parent
Sun et al. (2022) [100]	USA	online	longi	2016 - 2020	03. - nr.2020	247	4.5	247	53.4	7 - 9; 8.13 (.46)	child	parent
Syed et al. (2022) [101]	Bangladesh	online	cross		07. - 17.05.20	512	42.8	512	50.4	4 - 17; 8.6 (3.6)	both	parent
Thibodeau-Nielsen et al. (2021) [123]	USA	online	longi	05. - 09.2020	09.2020	99	4	99	49.5	3 - 6; 4.17 (0.75)	child	parent
Tso et al. (2020) [124]	Hong Kong	online	cross		03.2020	29202	nr	29202	51.4	2 - 12; 6.5 (2.84)	child	parent
Vet et al. (2021) [115]	Netherlands	online	cross		07.08. - 07.09.20	694	9.2	694	52	0 - 4; 2.26 (0.93)	child	parent
Wang et al. (2022) [102]	China	online	cross		05.2020	692	29.6	692	53.2	nr; 13.54 (.58)	adol	parent
Westrupp et al. (2021) [125]	Australia	online	cross		08. - 28.04.20	2365	19	2365	51	0 - 18; 8.66 (5.14)	both	parent
Yakşi et al. (2021) [126]	Turkey	online	cross		08.06. - 31.07.20	nr	nr	506	39.3	14 - 21; nr (nr)	adol	adol
Zambrana and Hart (2022) [134]	USA	online	longi	nr	nr	28	3	28	60.7	nr; 3.02 (.25)	child	parent
L. Zhang et al. (2022) [127]	USA	online + home visits	longi	01. - 10.2020	05. - 10.2020	124	7	124	48	nr; 12.89 (.79)	adol	both
X. Zhang (2022) [103]	China	paper-pencil	cross		06. - 07.2020	764	> 25.9	764	52.7	nr; 4.9 (1.02)	child	parent

Y. Zhang et al. (2022) [104]	China	online	cross		04. - 28.02.20	3471	43.6	3471	51.6	9 - 19; 14.1 (2.56)	adol	both
T. Zhou et al. (2022) [105]	China	online	cross		04.2020	5720	0	5720	51.1	10 - 17; 11.60 (1.36)	adol	both
X. Zhou et al. (2022) [106]	USA	online	longi	03. - 07.2020	30.03. - 14.04.20	488	33	488	56	3 - 8; 5.04 (1.59)	child	parent

adol adolescent, *cross* cross-sectional, *longi* longitudinal, *nr* not reported, *sw* whole sample, *s2* sample 2: toddlers.
