# Supplementary material for: Mental Health Is a Family Affair—Systematic Review and Meta-Analysis on the Associations between Mental Health Problems in Parents and Children during the COVID-19 Pandemic
Source: Int J Environ Res Public Health. 2023 Mar 2;20(5):4485. doi: 10.3390/ijerph20054485 (PMC10001622; doi:10.3390/ijerph20054485)
Supplement: Supplementary file 1 [file ijerph-20-04485-s001.zip › Table S3.pdf]

Table S3: Overview of included studies – variables and instruments.

Study	Children and Adolescents						Parents					
	Psycho-Pathology	Internalizing Symptoms	Depressive Symptoms	Anxiety Symptoms	Externalizing Symptoms	Stress	Prior Mental Illness	During COVID-19				
								Psycho-Pathology	Depressive Symptoms	Anxiety Symptoms	Stress	Parenting Stress
Achterberg et al. (2021) [61]						PSS		BSI (sf)			PSS	
Akgül and Atalan Ergin (2021) [62]				STAI						STAI		
Andrés-Romero et al. (2021) [128]	SDQ	SDQ (emot + peer)			SDQ (con + hyp)							ParSS
Babore et al. (2021) [28]			PROMIS					HADS				PSI (sf)
Berry et al. (2021) [107]					SDQ (con + hyp)						ahd-q	ParSS
Bianco et al. (2021) [61]	CBCL/6-18 (anx/dep + att + agg)	CBCL/6-18 (anx/dep)			CBCL/6-18 (att + agg)			DASS-21				
Black et al. (2021) [53]			MFQ-Child Self-Report (sf)				MFQ-Adult Self-Report (sf)		MFQ-Adult Self-Report (sf)			
Blackwell et al. (2022) [108]						ECHO ASDS					ECHO ASDS	
Borbás et al. (2021) [64]	SDQ							STAI-6 + CESD-R	CESD-R	STAI-6		BSFC-s
Browne et al. (2021) [65]	PROMIS							K10				
Büber and Aktaş Terzioğlu (2022) [109]	ahd-q	ahd-q			ahd-q						PSS	
Buechel et al. (2022) [66]	SDQ							STADI			ahd-i	PSI
Chan (2022) [110]						ahd-q					ahd-q	
Chartier et al. (2021)) [111]			PDI (des)			ahd-i					ahd-i	
Cohodes et al. (2021) [112]	CBCL/1.5-5 + CBCL/6-18	CBCL/1.5-5 (int) + CBCL/6-18 (int)			CBCL/1.5-5 (ext) + CBCL/6-18 (ext)						EPII	
Corbett et al. (2021) [113]						RSQ (child version)					RSQ (adult version)	
Crescentini et al. (2020) [67]		CBCL/6-18 (anx/dep + wit/dep + som)	CBCL/6-18 (wit/dep)					HADS				

Cusinato et al. (2020) [129]	SDQ											ParSS
Daks et al. (2020) [68]	CBCL (sel)								PHQ-9		ahd-q	
Davidson et al. (2021) [114]	SDQ										ESI	
Doan et al. (2022) [54]	CBCL	CBCL (int)			CBCL (ext)		BDI-II		BDI-II		ahd-q	
Dollberg and Hanetz-Gamliel (2022) [69]	CBCL 1.5-5 + CBCL 6-18	CBCL 1.5-5 (int) + CBCL 6-18 (int)			CBCL 1.5-5 (ext) + CBCL 6-18 (ext)			BSI (anx + dep)	BSI (dep)	BSI (anx)		
Donker et al. (2021) [116]						ahd-q					ahd-q	
Dubois-Comtois et al. (2021) [70]	YSR (int) + CBCL/6-18 (ext)	YSR (int)			CBCL/6-18 (ext)				BSI (sel)			
Essler et al. (2021) [117]	SDQ										ahd-q	
Feinberg et al. (2022) [71]	SDQ (sel)	SDQ (sel) (int)			SDQ (sel) (ext)			CES-D(sel) + PSWQ	CES-D (sel)	PSWQ		
Ferraro et al. (2021) [72]	SDQ							DASS-21	DASS-21(dep)	DASS-21 (anx)	DASS-21 (str)	
Feurer et al. (2021) [55]			CESD-R				BDI-II		BDI-II			USLA LSI (mot-child)
Fogarty et al. (2022) [56]		PHQ adol + GAD-7	PHQ adol	GAD-7			EPDS + CES-D + BAI + PCL-C		CES-D			
Foley et al. (2021) [73]	SDQ	SDQ (emot + peer)			SDQ (con + hyp)			GHQ-12				
Fong et al. (2021) [74]	Conners 3-P (global index)	Conners 3-P (anx + dep)	Conners 3-P (dep)	Conners 3-P (anx)	Conners 3-P (ina + hyp)	PSS child		DASS-21 (dep + anx)	DASS-21 (dep)	DASS-21 (anx)	PSS	
Fosco et al. (2022) [57]	ahd-q	ahd-q			ahd-q		CES-D + PSWQ (sf)					
Frigerio et al. (2022) [75]	CBCL/1.5-5 (emot-rea + anx/dep + som + wit/dep + att + agg)	CBCL/1.5-5 (emot-rea + anx/dep + som + wit/dep)			CBCL/1.5-5 (att + agg)			EPDS + STAI				
Giannotti et al. (2021) [130]					SDQ (ext)							ParSS (sel)
Giannotti et al. (2022) [131]					SDQ (ext)							ParSS (sel)
Glynn et al. (2021) [76]			PFC		SDQ (ext)				CES-D		PSS	

Hails et al. (2022) [77]	PPSC								PROMIS-29 (dep)			
Hollenstein et al. (2021) [58]		CDI-2 + BAI	CDI-2	BAI			BDI-II + BAI		BDI-II	BAI	EPII	
Khoury et al. (2021) [78]	BPM/6-18	BPM/6-18 (int)			BPM/6-18 (ext)			CES-D-10 + GAD-7	CES-D-10	GAD-7	PSS	
Kim et al. (2021) [79]	BPI								PHQ-9		ahd-i	
Köhler-Dauner et al. (2022) [59]	SDQ	SDQ (emot)			SDQ (con + hyp)		PHQ-9		PHQ-9			
Lee et al. (2021) [80]				CBCL/4-18 (anx)				PHQ-8 + GAD-7	PHQ-8	GAD-7		
Lengua et al. (2022) [60]	SDQ	SDQ (int)			SDQ (ext)		PHQ-9 + GAD-7		PHQ-9	GAD-7	ahd-q	
Li et al. (2021) [81]			PHQ-9						PHQ-9			
Liang et al. (2021) [118]		IS-COVID19 (sel)	IS-COVID19 (sel)	IS-COVID19 (sel)							PSS-10	
Lionetti et al. (2022) [132]	CBCL/1.5-5	CBCL/1.5-5 (int)			CBCL/1.5-5 (ext)							PSI-SF (PCDI subscale)
Low and Mounts (2022) [82]		RCADS						DASS-21				ParSS
Maggio et al. (2021) [83]		CDI + STAI child	CDI	STAI child				DASS-21	DASS-21 (dep)	DASS-21 (anx)	DASS-21 (str)	
Marchetti et al. (2020) [84]	SDQ (emot + hyp)	SDQ (emot)			SDQ (hyp)			GHQ-12				
Marzilli et al. (2021) [85]	SDQ							CPDI				PSI-SF
McArthur et al. (2021) [86]		BASC-3 (dep + anx)	BASC-3 (dep)	BASC-3 (anx)				CES-D-10 + SSAI-SF	CES-D-10	SSAI-SF		
McMahon et al. (2021) [87]	SDQ							DASS-21				
Mensi et al. (2021) [133]						PSS						PSI-SF (yr)
Morban et al. (2020) [88]		CBCL/1.5-5 (int)								BAI		
Moulin et al. (2021) [89]	SDQ (emot + hyp)	SDQ (emot)			SDQ (hyp)			ASR (anx/dep)				
Orgilés et al. (2021) [119]		SCAS par + MFQ par (sf)	SCAS par	MFQ par (sf)							ahd-i	
Penner et al. (2022) [90]	SDQ	SDQ (int)			SDQ (ext)			PROMIS			CEFIS	
Polónyiová et al. (2022) [91]	Vineland-3	Vineland-3 (int)			Vineland-3 (ext)			DASS-42	DASS-42 (dep)	DASS-42 (anx)	DASS-42 (str)	

Radanović et al. (2021) [92]				FSSC-R (sf, mod)					STICSA (trait version)		
Rizeq et al. (2021) [93]	RCADS + SCARED + CRISIS		RCADS	SCARED	CRISIS	CRISIS (subscale, yr)	PHQ-8 + GAD-7	PHQ-8	GAD-7	CRISIS (sel)	
Robertson et al. (2021) [94]	SDQ	SDQ (int)			SDQ (ext)		exp-cov19 (sel)			ESI	
Romero et al. (2020) [95]	SDQ (emot + con + hyp)	SDQ (emot)			SDQ (con + hyp)		PHQ-4	PHQ-4 (dep)	PHQ-4 (anx)	ahd-q	ahd-q
Russell et al. (2020) [96]						PSS child	MDI + GAD-7	MDI	GAD-7		BSFC-s
Russell et al. (2022) [120]						PSS child				dis-cov19 (mod)	
Saddik et al. (2021) [97]		SDQ (emot)							GAD-7		
Shelleby et al. (2022) [98]					BFS (ext) + SNAP-IV (hyp/imp + ODD)		DASS-21	DASS-21 (dep)	DASS-21 (anx)	DASS-21 (str)	
Singletary et al. (2022) [121]	PEDS (anx/wit + fear + act)	PEDS (anx/wit + fear)			PEDS (act)					ahd-q	
Spencer et al. (2021) [99]	PSC-17						PHQ-2 + GAD-2	PHQ-2	GAD-2		
Spinelli et al. (2020) [122]	SDQ (emot + con + hyp)		SDQ (emot)		SDQ (con + hyp)					DASS (sf, str)	PSI (sf)
Sun et al. (2022) [100]	PEDS (anx/wit + fear + act)	PEDS (anx/wit + fear)			PEDS (act)		CESDR-10 + GAD-7 + UCLA (lone) + BRS				
Syed et al. (2022) [101]	SDQ							PHQ-9			
Thibodeau-Nielsen et al. (2021) [123]		CBCL/1.5-5 (emot-rea + anx/dep)								PSS	ParSS (sf)
Tso et al. (2020) [124]	SDQ	SDQ (emot + peer)			SDQ (con + hyp)					PSS	
Vet et al. (2021) [115]						ahd-q				ahd-q	
Wang et al. (2022) [102]	SDQ						DASS-21				

Westrupp et al. (2021) [125]		SMFQ + SCAS par (sel)	SMFQ	SCAS par (sel)						CRISIS (mod)	
Yakşi et al. (2021) [126]		CDI + STAI	CDI	STAI						ahd-i	
Zambrana and Hart (2022) [134]	BASC-3	BASC-3 (int)			BASC-3 (ext)						PSI-SF
L. Zhang et al. (2022) [127]						CASPE				PSS	
X. Zhang (2022) [103]	PEDS	PEDS (anx/wit + fear)			PEDS (act)		CESD (sf) + GAD-7	CESD (sf)	GAD-7		
Y. Zhang et al. (2022) [104]		PHQ-9 + GAD-7	PHQ-9	GAD-7			PHQ-9 + GAD-7	PHQ-9	GAD-7		
T. Zhou et al. (2022) [105]		CBCL/4-18 (anx/dep)							GAD-7		
X. Zhou et al. (2022) [106]	PEDS								GAD-7		

act acting out behaviors subscale, *adol* adolescents, *agg* aggressive behavior subscale, *ahd-i* ad hoc developed single item, *ahd-q* ad hoc developed questionnaire, *anx* anxiety subscale, *anx/dep* anxious-depressed subscale, *anx/wit* anxious and withdrawn behaviors subscale, *ASR* Adult Self Report Scale, *att* attention problems subscale, *BAI* Beck Anxiety Inventory, *BASC-3* Behavior Assessment System for Children, *BDI-II* Beck Depression Inventory II, *BFS* Behavior and Feeling Survey, *BPI* Behavior Problem Index, *BPM* Brief Problem Monitor - Parent form, *BRS* Brief Resilience Scale, *BSI* Brief Symptom Inventory, *BSFC-s* short version of the Burden Scale for Family Caregivers, *CASPE* COVID-19 Adolescent Symptom & Psychological Experience Questionnaire, *CBCL* Child Behavior Checklist, *CDI* Children's Depression Inventory, *CEFIS* COVID-19 Exposure of the Family Impact Survey, *CESD* Center for Epidemiologic Studies Depression Scale, *CESD-R* Center for Epidemiologic Studies Depression Scale Revised, *child* children, *con* conduct problems subscale, *Conners 3-P* Conners 3rd edition Parent version, *CPDI* COVID-19 Peritraumatic Distress Index, *CRISIS* CoRonavIruS Health Impact Survey, *DASS* Depression-Anxiety-Stress-Scale, *dep* depression subscale, *des* dysphoric emotions subscale, *dis-cov19* Disaster-specific measure of COVID-19 stressors, *ECHO ASDS* Environmental influences on Child Health Outcomes - Acute Stress Disorder Scale, *emot* emotional problems subscale, *emot-rea* emotionally reactive subscale, *EPDS* Edinburgh Postnatal Depression Scale, *EPII* Epidemic-Pandemic Impacts Inventory, *ESI* Everyday Stressors Index, *ext* externalizing problems subscale, *exp-cov19* Experiences Related to COVID-19 Questionnaire, *fear* fearful behaviors subscale, *FSSC-R* Fear Schedule for Children Revised, *GAD* Generalized Anxiety Disorder Scale, *GHQ* General Health Questionnaire, *HADS* Hospital Anxiety and Depression Scale, *hyp* hyperactivity subscale, *hyp/imp* hyperactivity/impulsivity subscale, *ina* inattention subscale, *int* internalizing problems subscale, *IS-COVID19* Impact Scale of COVID-19 and home confinement on children and adolescents, *K10* Kessler Psychological Distress Scale, *lone* loneliness subscale, *LSI* Life Stress Interview, *MFQ* Mood and Feelings Questionnaire, *mod* modified, *mot-child* mother child subscale, *par* parents, *ParSS* Parental Stress Scale, *PCDI* Parent Child Dysfunctional Interaction scale, *PCL-C* Post-Traumatic Stress Disorder Checklist - Civilian version, *PDI* Peritraumatic Distress Inventory, *PEDS* Pediatric Emotional Distress Scale, *peer* peer problems subscale, *PFC* PreSchool Feelings Checklist, *PHQ* Patient Health Questionnaire, *PPSC* Preschool Pediatric Symptom Checklist, *PROMIS* – Emotional Distress-Depression-Pediatric Item Bank, *PSC* Pediatric Symptom Checklist, *PSI* Parenting Stress Index, *PSS* Perceived Stress Scale, *PSSC* Perceived Stress Scale Child, *PSWQ* Penn State Worry Questionnaire, *RCADS* Revised Child Anxiety and Depression Scale, *RSQ* Responses to Stress Questionnaire, *SCARED* Screen for Child Anxiety-Related Disorders, *SCAS-Parent* Spence Children's Anxiety scale Parent version, *SDQ* Strengths and Difficulties Questionnaire, *sel* selection of items, *sf* short form, *SMFQ* Short Mood and Feelings Questionnaire, *SNAP-IV* Swanson, Nolan and Pelham rating scale, *som* somatic complaints subscale, *SSAI-SF* Spielberger State Anxiety Inventory - Short Form, *STADI* State-Trait-Anxiety-Depression Inventory, *STAI* State Trait Anxiety Inventory, *STICSA* Trait version of the State - Trait Inventory for Cognitive and Somatic Anxiety, *str* stress subscale, *Vineland-3* Vineland Adaptive Behavior Scales - Third Edition, *wit/dep* withdrawn-depressed subscale, *yr* youth report.
