# Supplementary material for: Mental Health Is a Family Affair—Systematic Review and Meta-Analysis on the Associations between Mental Health Problems in Parents and Children during the COVID-19 Pandemic
Source: Int J Environ Res Public Health. 2023 Mar 2;20(5):4485. doi: 10.3390/ijerph20054485 (PMC10001622; doi:10.3390/ijerph20054485)
Supplement: Supplementary file 1 [file ijerph-20-04485-s001.zip › Table S4.pdf]

Table S4: Quality assessment of included studies.

Study		Achterberg et al. (2021) [61]	Akgül and Atalan Ergin (2021) [62]	Andrés-Romero et al. (2021) [128]	Babore et al. (2021) [28]	Berry et al. (2021) [107]	Bianco et al. (2021) [61]	Black et al. (2021) [53]	Blackwell et al. (2022) [108]	Borbás et al. (2021) [64]	Browne et al. (2021) [65]
**AXIS overall rating**		**16/20**	**15/20**	**15/20**	**16/20**	**15/20**	**15/20**	**15/20**	**15/20**	**15/20**	**16/20**
**Risk of Bias**		**medium**	**medium**	**medium**	**medium**	**medium**	**medium**	**medium**	**medium**	**medium**	**medium**
1	aims/objectives clear?	Yes	yes	yes	yes	yes	yes	yes	yes	yes	yes
2	study design appropriate?	Yes	yes	yes	yes	yes	yes	yes	yes	yes	yes
3	sample size justified?	No	yes	no	yes	yes	no	no	no	no	no
4	target population clearly defined?	Yes	yes	yes	yes	yes	yes	yes	yes	yes	yes
5	sample frame appropriate?	No	no	yes	yes	yes	yes	yes	yes	no	yes
6	process selects representative sample	Yes	yes	yes	yes	yes	yes	yes	yes	yes	yes
7	non-responders addressed?	Yes	no	no	no	no	no	no	no	no	no
8	appropriate outcome variables?	Yes	yes	yes	yes	yes	yes	yes	yes	yes	yes
9	valid and reliable outcome measures?	No	no	no	yes	no	yes	yes	no	no	yes
10	statistical significance clear?	Yes	yes	yes	yes	yes	yes	yes	yes	yes	yes
11	methods sufficiently described?	Yes	yes	yes	yes	yes	yes	yes	yes	yes	yes
12	basic data adequately described?	Yes	yes	yes	yes	yes	yes	yes	yes	yes	yes
13	response rate raises concerns about non-response bias?	No	don't know	don't know	don't know	don't know	don't know	don't know	don't know	no	don't know
14	non-responders described?	Yes	no	no	no	no	no	no	no	no	no
15	results internally consistent?	No	yes	yes	no	no	no	no	yes	yes	yes
16	all results presented?	Yes	yes	yes	yes	yes	yes	yes	yes	yes	yes
17	conclusions justified by results?	Yes	yes	yes	yes	yes	yes	yes	yes	yes	yes

18	limitations discussed?	Yes	yes	yes	yes	yes	yes	yes	yes	yes	yes
19	Conflict of interest?	No	no	no	no	no	no	no	no	no	no
20	ethical approval or consent?	Yes	yes	yes	yes	yes	yes	yes	yes	yes	yes
comments		3 nr, 5 sfols-nfi, 9 pum, pam, 15 N inconsistent	5 rec restricted, 7 nr, 9 pam, 13 rr nr, 14 nr	3 nr, 7 nr, 9 pum, 13 rr nr, 14 nr	7 nr, 13 rr nr, 14 nr, 15 difi t1	7 nr, 9 pum, 13 rr nr, 1 nr, 15 difi	3 nr, 7 nr, 13 rr nr, 14 nr, 15 p difi	3 nr, 7 nr, 13 rr nr, 14 nr, 15 difi f1	3 nr, 7 nr, 9 pum or psim, 13 rr nr, 14 nr	3 nr, 5 sfols-nfi, 7 nr, 9 pum, pam, 13 rr = 70 %, 14 nr	3 nr, 7 nr, 13 rr nr, 14 nr

coi conflict of interest declared, *difi (tx/fx)* partly information differ between text (and table x/figure x), *fnd* funding, *fnd-coi nr* external funding-but potential conflicts of interest not declared, *nr* not reported, *pam* partly adapted measurements, *pBD nr* not all basic date reported, *psim* partly single item measurements, *pum* partly unvalidated measurements, *rec* recruitment, *rel* reliabilities, *rr* response rate, *sfols-nfi* sample from ongoing longitudinal study-no further information given, *sw* software, *tot ≠ 100 (tx)* numbers don't add up to 100 % / N (in table x)

Study		Büber and Aktaş Terzioğlu (2022) [109]	Buechel et al. (2022) [66]	Chan (2022) [110]	Chartier et al. (2021) [111]	Cohodes et al. (2021) [112]	Corbett et al. (2021) [113]	Crescentini et al. (2020) [67]	Cusinato et al. (2020) [129]	Daks et al. (2020) [68]	Davidson et al. (2021) [114]
**AXIS overall rating**		**15/20**	**14/20**	**14/20**	**12/20**	**15/20**	**17/20**	**15/20**	**15/20**	**16/20**	**13/20**
**Risk of Bias**		**medium**	**medium**	**medium**	**high**	**medium**	**medium**	**medium**	**medium**	**medium**	**high**
1	aims/objectives clear?	yes	yes	yes	yes	yes	yes	yes	yes	yes	yes
2	study design appropriate?	yes	yes	yes	yes	yes	yes	yes	yes	yes	yes
3	sample size justified?	yes	no	no	no	yes	no	no	yes	yes	no
4	target population clearly defined?	yes	yes	yes	yes	yes	yes	yes	yes	yes	yes
5	sample frame appropriate?	yes	yes	yes	yes	yes	yes	yes	no	yes	no
6	process selects representative sample	yes	yes	yes	yes	yes	yes	yes	yes	yes	no
7	non-responders addressed?	no	no	no	no	no	yes	no	no	no	no
8	appropriate outcome variables?	yes	yes	yes	yes	yes	yes	yes	yes	yes	yes
9	valid and reliable outcome measures?	no	no	no	no	no	yes	no	no	no	no
10	statistical significance clear?	yes	yes	yes	no	no	no	yes	yes	yes	yes
11	methods sufficiently described?	yes	yes	yes	yes	yes	yes	yes	yes	yes	yes
12	basic data adequately described?	yes	no	yes	no	yes	yes	yes	yes	yes	yes
13	response rate raises concerns about non-response bias?	yes	yes	don't know	don't know	don't know	no	don't know	don't know	don't know	yes
14	non-responders described?	no	no	no	no	no	yes	no	no	no	no
15	results internally consistent?	no	yes	no	yes	yes	no	yes	yes	yes	yes
16	all results presented?	yes	yes	yes	yes	yes	yes	yes	yes	yes	yes
17	conclusions justified by results?	yes	yes	yes	yes	yes	yes	yes	yes	yes	yes
18	limitations discussed?	yes	yes	yes	yes	yes	yes	yes	yes	yes	yes

19	Conflict of interest?	no	no	no	no	no	no	no	no	no	no
20	ethical approval or consent?	yes	yes	yes	no	yes	yes	yes	yes	yes	yes
comments		7 nr, 9 pum, psim, 13 rr 23 %, 14 nr, 15 difi t1	3 nr, 7 nr, 9 pum, 12 N nr, SD nr, 13 rr ~ 20 %, 14 nr	3 nr, 9 pum, 13 rr nr, 14 nr, 15 difi	3 nr, 7 nr, 9 pum, psim, 10 sw nr, 12 pBD nr, 13 rr nr, 14 nr, 20 nr	7 nr, 9 pam, 10 sw nr, 13 rr nr, 14 nr	3 nr, 10 sw nr, 15 difi t2	3 nr, 7 nr, 9 pam, 13 rr nr, 14 nr	5 rec unclear, 7 nr, 9 pum, 13 rr nr, 14 nr	7 nr, 9 pum, pam, 13 rr nr, 14 nr	3 nr, 5 rec restricted, 6 selection not anonymously, 7 nr, 9 pum, 13 rr ~ 13 %, 14 nr

Study		Doan et al. (2022) [54]	Dollberg and Hanetz-Gamliel (2022) [69]	Donker et al. (2021) [116]	Dubois-Comtois et al. (2021) [70]	Essler et al. (2021) [117]	Feinberg et al. (2022) [71]	Ferraro et al. (2021) [72]	Feurer et al. (2021) [55]	Fogarty et al. (2022) [56]	Foley et al. (2021) [73]
**AXIS overall rating**		**14/20**	**17/20**	**15/20**	**15/20**	**16/20**	**14/20**	**14/20**	**16/20**	**15/20**	**12/20**
**Risk of Bias**		**medium**	**medium**	**medium**	**medium**	**medium**	**medium**	**medium**	**medium**	**medium**	**high**
1	aims/objectives clear?	yes	yes	yes	yes	yes	yes	yes	yes	yes	yes
2	study design appropriate?	yes	yes	yes	yes	yes	yes	yes	yes	yes	yes
3	sample size justified?	no	yes	yes	no	yes	no	no	no	no	no
4	target population clearly defined?	yes	yes	yes	yes	yes	yes	yes	yes	yes	yes
5	sample frame appropriate?	yes	yes	yes	yes	yes	no	yes	yes	yes	yes
6	process selects representative sample	yes	yes	yes	yes	yes	yes	yes	yes	yes	yes
7	non-responders addressed?	no	no	no	no	no	yes	no	no	yes	no
8	appropriate outcome variables?	yes	yes	yes	yes	yes	yes	yes	yes	yes	yes
9	valid and reliable outcome measures?	no	yes	no	yes	no	no	no	yes	no	no
10	statistical significance clear?	yes	yes	yes	yes	yes	yes	yes	yes	no	yes
11	methods sufficiently described?	yes	yes	yes	yes	yes	yes	yes	yes	yes	yes
12	basic data adequately described?	yes	yes	yes	yes	yes	yes	yes	yes	yes	no
13	response rate raises concerns about non-response bias?	yes	don't know	don't know	don't know	don't know	no	don't know	don't know	yes	don't know
14	non-responders described?	no	no	no	no	no	yes	no	no	yes	no
15	results internally consistent?	no	yes	yes	no	yes	no	no	yes	yes	no
16	all results presented?	yes	yes	yes	yes	yes	yes	yes	yes	yes	yes
17	conclusions justified by results?	yes	yes	yes	yes	yes	yes	yes	yes	yes	no
18	limitations discussed?	yes	yes	yes	yes	yes	yes	yes	yes	yes	yes

19	Conflict of interest?	no	no	don't know	no	no	don't know	no	no	yes	no
20	ethical approval or consent?	yes	yes	yes	yes	yes	don't know	yes	yes	yes	yes
comments		3 nr, 7 nr, 9 pum, 13 higher depression rates in responders- possible response bias, 14 nr, 15 difi	7 nr, 13 rr nr, 14 nr	7 nr, 9 pum, 13 rr nr, 14 nr, 19 nr	3 nr, 7 nr, 13 rr nr, 14 nr, 15 difi	7 nr, 9 pam, psim, 13 rr nr, 14 nr	3 nr, 5 sfols-nfi, 9 pam, 15 difi t, 19 coi nr, 20 nr	3 nr, 7 nr, 9 pum, 13 rr nr, 14 nr, 15 difi f6	3 posthoc power analysis, but underpowered for small effects, 7 nr, 13 rr nr, 14 nr	3 nr, 9 pum, 10 sw nr, 13 rr < 30 %, 19 fnd-coi nr	3 nr, 7 nr, 9 pum, psim, 12 M nr, SD nr, inconsistent age range, 13 rr nr, 14 nr, 15 difi, 17 discussion partly not supported by results

Study		Fong et al. (2021) [74]	Fosco et al. (2022) [57]	Frigerio et al. (2022) [75]	Giannotti et al. (2021) [130]	Giannotti et al. (2022) [131]	Glynn et al. (2021) [76]	Hails et al. (2022) [77]	Hollenstein et al. (2021) [58]	Khoury et al. (2021) [78]	Kim et al. (2021) [79]
**AXIS overall rating**		**15/20**	**17/20**	**18/20**	**14/20**	**15/20**	**17/20**	**17/20**	**19/20**	**19/20**	**14/20**
**Risk of Bias**		**medium**	**medium**	**low**	**medium**	**medium**	**medium**	**medium**	**low**	**low**	**medium**
1	aims/objectives clear?	yes	yes	yes	yes	yes	yes	yes	yes	yes	yes
2	study design appropriate?	yes	yes	yes	yes	yes	yes	yes	yes	yes	yes
3	sample size justified?	no	no	no	no	no	no	yes	yes	no	no
4	target population clearly defined?	yes	yes	yes	yes	yes	yes	yes	yes	yes	yes
5	sample frame appropriate?	yes	yes	yes	yes	yes	yes	yes	yes	yes	no
6	process selects representative sample	yes	yes	yes	yes	yes	yes	yes	yes	yes	yes
7	non-responders addressed?	no	yes	yes	no	yes	yes	no	yes	yes	no
8	appropriate outcome variables?	yes	yes	yes	yes	yes	yes	yes	yes	yes	yes
9	valid and reliable outcome measures?	yes	no	yes	no	no	no	yes	yes	yes	no
10	statistical significance clear?	yes	yes	yes	no	yes	yes	yes	yes	yes	yes
11	methods sufficiently described?	yes	yes	yes	yes	yes	yes	yes	yes	yes	yes
12	basic data adequately described?	yes	yes	no	yes	yes	yes	yes	yes	yes	yes
13	response rate raises concerns about non-response bias?	don't know	no	no	don't know	don't know	no	yes	no	no	don't know
14	non-responders described?	no	yes	yes	no	no	yes	no	yes	yes	no
15	results internally consistent?	no	yes	yes	yes	no	yes	yes	yes	yes	yes
16	all results presented?	yes	yes	yes	yes	yes	yes	yes	yes	yes	yes
17	conclusions justified by results?	yes	yes	yes	yes	yes	yes	yes	yes	yes	yes
18	limitations discussed?	yes	yes	yes	yes	yes	yes	yes	yes	yes	yes
19	Conflict of interest?	no	no	no	no	no	no	no	don't know	no	no

20	ethical approval or consent?	yes	don't know	yes	yes	yes	don't know	yes	yes	yes	yes
comments		3 nr, 7 nr, 13 rr nr, 14 nr, 15 difi	3 nr, 9 pam, 20 nr	3 nr, 12 M nr, SD nr	3 nr, 7 nr, 9 pam, 10 sw nr, 13 rr nr, 14 nr	3 nr, 9 pam, 13 rr nr, 14 nr, 15 difi	3 nr, 9 pam, 20 nr	3 nr, 7 nr, 13 rr = 16 %, 14 nr	19 nr	3 nr	3 nr, 5 rec unclear, 7 nr, 9 pum, psim, 13 rr nr, 14 nr

Study		Köhler-Dauner et al. (2022) [59]	Lee et al. (2021) [80]	Lengua et al. (2022) [60]	Li et al. (2021) [81]	Liang et al. (2021) [118]	Lionetti et al. (2022) [132]	Low and Mounts (2022) [82]	Maggio et al. (2021) [83]	Marchetti et al. (2020) [84]	Marzilli et al. (2021) [85]
**AXIS overall rating**		**17/20**	**15/20**	**19/20**	**13/20**	**15/20**	**14/20**	**15/20**	**13/20**	**15/20**	**12/20**
**Risk of Bias**		**medium**	**medium**	**low**	**high**	**medium**	**medium**	**medium**	**high**	**medium**	**high**
1	aims/objectives clear?	yes	yes	yes	yes	yes	yes	yes	yes	yes	yes
2	study design appropriate?	yes	yes	yes	yes	yes	yes	yes	yes	yes	yes
3	sample size justified?	no	no	yes	no	no	no	no	no	no	no
4	target population clearly defined?	yes	yes	yes	yes	yes	yes	yes	yes	yes	yes
5	sample frame appropriate?	yes	yes	don't know	no	yes	don't know	yes	yes	yes	yes
6	process selects representative sample	yes	yes	yes	no	yes	yes	yes	yes	yes	yes
7	non-responders addressed?	no	no	yes	no	no	no	no	no	no	no
8	appropriate outcome variables?	yes	yes	yes	yes	yes	yes	yes	no	yes	yes
9	valid and reliable outcome measures?	yes	no	yes	yes	no	yes	yes	no	no	no
10	statistical significance clear?	yes	yes	yes	yes	yes	yes	yes	yes	yes	yes
11	methods sufficiently described?	yes	yes	yes	yes	yes	yes	yes	yes	yes	yes
12	basic data adequately described?	yes	yes	yes	yes	yes	yes	yes	yes	yes	no
13	response rate raises concerns about non-response bias?	no	don't know	no	don't know	don't know	no	don't know	don't know	don't know	don't know
14	non-responders described?	no	no	yes	no	no	no	no	no	no	no
15	results internally consistent?	yes	yes	yes	no	yes	no	yes	yes	yes	no
16	all results presented?	yes	yes	yes	yes	yes	yes	yes	no	yes	yes
17	conclusions justified by results?	yes	yes	yes	yes	yes	yes	yes	yes	yes	no
18	limitations discussed?	yes	yes	yes	yes	yes	yes	yes	yes	yes	yes
19	Conflict of interest?	no	no	no	no	no	no	don't know	no	no	no

20	ethical approval or consent?	yes	yes	yes	yes	yes	don't know	yes	yes	yes	yes
comments		3 nr, 13 rr = 60 %, 14 nr, 15 difi	3 nr, 7 nr, 9 pam, 13 rr nr, 14 nr	5 sfols-nfi, 13 rr = 50 %	3 nr, 5 rec unclear, 6 inclusion/exclusion criteria nr, 7 nr, 13 rr nr, 14 nr, 15 difi t1	3 nr, 7 nr, 9 pum, pam, 13 rr nr, 14 nr	3 nr, 5 sfols-nfi, 7 no, 13 rr = 86 %, 14 nr, 15 difi, 20 nr	3 nr, 7 nr, 13 rr nr, 14 nr, 19 nr	3 nr, 7 nr, 8 pum for age of participants, 9 pum, 13 rr nr, 14 nr, 16 value of correlations nr	3 nr, 7 nr, 9 pam, 13 rr nr, 14 nr	3 nr, 7 nr, 9 pum, 12 M nr, SD nr, 13 rr nr, 14 nr, 15 difi, 17 discussion partly not supported by results

Study		McArthur et al. (2021) [86]	McMahon et al. (2021) [87]	Mensi et al. (2021) [133]	Morban et al. (2020) [88]	Moulin et al. (2021) [89]	Orgilés et al. (2021) [119]	Penner et al. (2022) [90]	Polónyiová et al. (2022) [91]	Radanović et al. (2021) [92]	Rizeq et al. (2021) [93]
**AXIS overall rating**		**14/20**	**14/20**	**14/20**	**13/20**	**16/20**	**15/20**	**16/20**	**14/20**	**15/20**	**14/20**
**Risk of Bias**		**medium**	**medium**	**medium**	**high**	**medium**	**medium**	**medium**	**medium**	**medium**	**medium**
1	aims/objectives clear?	yes	yes	yes	yes	yes	yes	yes	yes	yes	yes
2	study design appropriate?	yes	yes	yes	yes	yes	yes	yes	yes	yes	yes
3	sample size justified?	no	no	no	no	no	no	no	no	no	no
4	target population clearly defined?	yes	yes	yes	yes	yes	yes	yes	yes	yes	yes
5	sample frame appropriate?	yes	yes	yes	no	yes	yes	yes	yes	yes	no
6	process selects representative sample	yes	yes	yes	yes	yes	yes	yes	yes	yes	yes
7	non-responders addressed?	no	no	no	no	no	no	no	no	no	no
8	appropriate outcome variables?	yes	yes	yes	yes	yes	yes	yes	yes	yes	yes
9	valid and reliable outcome measures?	no	no	no	no	no	no	yes	no	no	no
10	statistical significance clear?	yes	yes	yes	yes	yes	yes	yes	yes	yes	yes
11	methods sufficiently described?	yes	yes	yes	yes	yes	yes	yes	yes	yes	yes
12	basic data adequately described?	yes	yes	yes	no	yes	yes	yes	yes	yes	yes
13	response rate raises concerns about non-response bias?	don't know	don't know	don't know	yes	no	don't know	no	don't know	don't know	don't know
14	non-responders described?	no	no	no	no	no	no	no	no	no	no
15	results internally consistent?	no	no	yes	yes	yes	yes	yes	no	yes	yes
16	all results presented?	yes	yes	no	yes	yes	yes	yes	yes	yes	yes
17	conclusions justified by results?	yes	yes	yes	yes	yes	yes	yes	yes	yes	yes
18	limitations discussed?	yes	yes	yes	yes	yes	yes	yes	yes	yes	yes
19	Conflict of interest?	no	no	no	no	no	no	yes	no	no	no

20	ethical approval or consent?	yes	yes	yes	yes	yes	yes	yes	yes	yes	yes
comments		3 nr, 7 nr, 9 pum, psim, pam, 13 rr nr, 14 nr, 15 difi t2	3 nr, 7 nr, 9 pum, psim, 13 rr nr, 14 nr, 15 tot ≠ 100, difi t2	3 nr, 7 nr, 9 pam, reliabilities nr, 13 rr nr, 14 nr, 16 results not described in detail	3 nr, 5 rec unclear, 7 nr, 9 pam, 12 pBD nr, 13 rr nr, very small sample size (N = 29), 14 nr	3 nr, 7 nr, 9 pum, pam, 14 nr	3 nr, 7 nr, 9 pum, 13 rr nr, 14 nr	3 nr, 14 nr, 19 coi	3 nr, 7 nr, 9 partly information about questionnaires nr, 13 rr nr, 14 nr, 15 difi	3 nr, 7 nr, 9 pum, pam, 13 rr nr, 14 nr	3 nr, 5 rec selective, 7 nr, 9 pam

Study		Robertson et al. (2021) [94]	Romero et al. (2020) [95]	Russell et al. (2020) [96]	Russell et al. (2022) [120]	Saddik et al. (2021) [97]	Shelleby et al. (2022) [98]	Singletary et al. (2022) [121]	Spencer et al. (2021) [99]	Spinelli et al. (2020) [122]	Sun et al. (2022) [100]
**AXIS overall rating**		**15/20**	**14/20**	**15/20**	**15/20**	**15/20**	**13/20**	**14/20**	**16/20**	**14/20**	**17/20**
**Risk of Bias**		**medium**	**medium**	**medium**	**medium**	**medium**	**high**	**medium**	**medium**	**medium**	**medium**
1	aims/objectives clear?	yes	yes	yes	yes	yes	yes	yes	yes	yes	yes
2	study design appropriate?	yes	yes	yes	yes	yes	yes	yes	yes	yes	yes
3	sample size justified?	yes	no	no	no	yes	no	no	no	no	no
4	target population clearly defined?	yes	yes	yes	yes	yes	yes	yes	yes	yes	yes
5	sample frame appropriate?	yes	yes	yes	yes	yes	yes	yes	yes	yes	yes
6	process selects representative sample	no	yes	yes	yes	yes	yes	yes	yes	no	yes
7	non-responders addressed?	no	no	no	no	no	no	no	yes	no	yes
8	appropriate outcome variables?	yes	yes	yes	yes	yes	yes	yes	yes	yes	yes
9	valid and reliable outcome measures?	no	no	yes	no	no	no	no	no	no	yes
10	statistical significance clear?	yes	yes	yes	yes	yes	yes	yes	yes	yes	yes
11	methods sufficiently described?	yes	yes	yes	yes	yes	yes	yes	yes	yes	yes
12	basic data adequately described?	yes	yes	yes	yes	yes	yes	yes	yes	yes	yes
13	response rate raises concerns about non-response bias?	yes	don't know	don't know	don't know	don't know	don't know	no	yes	don't know	no
14	non-responders described?	no	no	no	no	no	no	no	yes	no	no
15	results internally consistent?	yes	no	yes	yes	no	no	no	no	yes	no
16	all results presented?	yes	yes	yes	yes	yes	yes	yes	yes	yes	yes
17	conclusions justified by results?	yes	yes	yes	yes	yes	yes	yes	yes	yes	yes
18	limitations discussed?	yes	yes	yes	yes	yes	yes	yes	yes	yes	yes
19	Conflict of interest?	no	no	don't know	no	no	don't know	don't know	no	no	no

20	ethical approval or consent?	yes	yes	yes	yes	yes	yes	yes	yes	yes	yes
comments		6 selection not anonymously, 7 nr, 9 pam, 13 rr ~ 15 %, 14 nr	3 nr, 7 nr, 9 pum, 13 rr nr, 14 nr, 15 BD reported inconsistently	3 nr, 7 nr, 13 rr nr, 14 nr, 19 nr	3 nr, 7 nr, 9 pum, 13 rr nr, 14 nr	7 nr, 9 pum, 13 rr nr, 14 nr, 15 tot ≠ 100	3 nr, 7 nr, 9 pum, 13 rr nr, 14 nr, 15 difi, 19 nr	3 nr, 7 nr, 9 pum, 13 rr ~ 60 %, 14 nr, 15 difi, 19 fnd-coi nr	3 nr, 9 pum, 13 rr = 15 %, 15 tot ≠ 100	3 nr, 6 short recruitment period (5 days), 7 nr, 9 pum, pam, 13 rr nr, 14 nr	3 nr, 13 rr = 80 %, 14 nr

Study		Syed et al. (2022) [101]	Thibodeau-Nielsen et al. (2021) [123]	Tso et al. (2020) [124]	Vet et al. (2021) [115]	Wang et al. (2022) [102]	Westrupp et al. (2021) [125]	Yakşi et al. (2021) [126]	Zambrana and Hart (2022) [134]	L. Zhang et al. (2022) [127]	X. Zhang (2022) [103]
**AXIS overall rating**		**13/20**	**14/20**	**16/20**	**16/20**	**15/20**	**17/20**	**16/20**	**15/20**	**13/20**	**16/20**
**Risk of Bias**		**high**	**medium**	**medium**	**medium**	**medium**	**medium**	**medium**	**medium**	**high**	**medium**
1	aims/objectives clear?	yes	yes	yes	yes	yes	yes	yes	yes	yes	yes
2	study design appropriate?	yes	yes	yes	yes	yes	yes	yes	yes	yes	yes
3	sample size justified?	yes	no	no	yes	yes	no	no	no	no	no
4	target population clearly defined?	yes	yes	yes	yes	yes	yes	yes	yes	yes	yes
5	sample frame appropriate?	don't know	yes	yes	yes	no	yes	yes	yes	don't know	yes
6	process selects representative sample	yes	yes	yes	yes	yes	yes	yes	yes	yes	yes
7	non-responders addressed?	no	no	no	no	no	yes	no	no	no	no
8	appropriate outcome variables?	yes	yes	yes	yes	yes	yes	yes	yes	yes	yes
9	valid and reliable outcome measures?	yes	no	no	no	no	no	no	yes	yes	no
10	statistical significance clear?	yes	no	yes	yes	yes	yes	yes	no	yes	yes
11	methods sufficiently described?	no	yes	yes	yes	yes	yes	yes	yes	yes	yes
12	basic data adequately described?	no	yes	yes	yes	yes	yes	yes	yes	yes	yes
13	response rate raises concerns about non-response bias?	don't know	don't know	no	no	don't know	don't know	no	yes	don't know	no
14	non-responders described?	no	no	no	no	no	yes	no	no	no	no
15	results internally consistent?	yes	yes	yes	no	yes	yes	yes	yes	no	yes
16	all results presented?	don't know	yes	yes	yes	yes	yes	yes	yes	yes	yes
17	conclusions justified by results?	yes	yes	yes	yes	yes	yes	yes	yes	yes	yes
18	limitations discussed?	yes	yes	yes	yes	yes	yes	yes	yes	yes	yes
19	Conflict of interest?	no	no	no	no	no	no	no	no	don't know	no

20	ethical approval or consent?	yes	yes	yes	yes	yes	yes	yes	yes	yes	yes
comments		7 nr, 11 analysis plan nr, 12 M nr, SD nr, 13 rr nr, 14 nr, 16 analysis plan nr	3 nr, 7 nr, 9 pam, 10 sw nr, 13 rr nr, 14 nr	3 nr, 7 nr, 9 rel nr, 14 nr	7 nr, 9 pum, 14 nr, 15 difi	5 rec restricted, 7 nr, 9 pum, 13 rr nr, 14 nr	3 nr, 9 pam, 13 rr nr	3 underpowered, 7 nr, 9 pum, psim, 13 rr = 38 %, 14 nr	3 nr, 7 nr, 10 sw nr, 13 N only 28, 14 nr	3 nr, 5 sfols-nfi, 13 rr nr, 14 nr, 15 difit, 19 fnd-coi nr	3 nr, 7 nr, 9 pum, 13 rr > 60 %, 14 nr

**Study**		Y. Zhang et al. (2022) [104]	T. Zhou et al. (2022) [105]	X. Zhou et al. (2022) [106]
**AXIS overall rating**		**16/20**	**15/20**	**14/20**
**Risk of Bias**		**medium**	**medium**	**medium**
1	aims/objectives clear?	yes	yes	yes
2	study design appropriate?	yes	yes	yes
3	sample size justified?	no	no	no
4	target population clearly defined?	yes	yes	yes
5	sample frame appropriate?	yes	yes	yes
6	process selects representative sample	yes	yes	yes
7	non-responders addressed?	no	no	no
8	appropriate outcome variables?	yes	yes	yes
9	valid and reliable outcome measures?	no	no	yes
10	statistical significance clear?	yes	yes	yes
11	methods sufficiently described?	yes	yes	yes
12	basic data adequately described?	yes	yes	yes
13	response rate raises concerns about non-response bias?	no	don't know	don't know
14	non-responders described?	no	no	no
15	results internally consistent?	yes	yes	yes
16	all results presented?	yes	yes	yes
17	conclusions justified by results?	yes	yes	yes
18	limitations discussed?	yes	yes	yes
19	Conflict of interest?	no	no	don't know
20	ethical approval or consent?	yes	yes	don't know
comments		3 nr, 7 nr, 9 pum, 13 rr ~ 50 %, 14 nr	3 nr, 7 nr, 9 pum, 13 rr nr, 14 nr	3 nr, 7 nr, 13 rr nr, 14 nr, 19 nr, 20 nr
